# A National Survey Assessing SARS-CoV-2 Vaccination Intentions: Implications for Future Public Health Communication Efforts

**DOI:** 10.1177/1075547020960463

**Published:** 2020-10

**Authors:** Katharine J. Head, Monica L. Kasting, Lynne A. Sturm, Jane A. Hartsock, Gregory D. Zimet

**Affiliations:** 1 Indiana University–Purdue University Indianapolis, Indianapolis, IN, USA; 2 Purdue University, West Lafayette, IN, USA; 3 Indiana University School of Medicine, Indianapolis, IN, USA

**Keywords:** vaccination intentions, COVID-19, SARS-CoV-2, perceived threat, provider recommendation

## Abstract

With SARS-CoV-2 vaccines under development, research is needed to assess intention to vaccinate. We conducted a survey (*N* = 3,159) with U.S. adults in May 2020 assessing SARS-CoV-2 vaccine intentions, intentions with a provider recommendation, and sociodemographic and psychosocial variables. Participants had high SARS-CoV-2 vaccine intentions (*M* = 5.23/7-point scale), which increased significantly with a provider recommendation (*M* = 5.47). Hierarchical linear regression showed that less education and working in health care were associated with lower intent, and liberal political views, altruism, and COVID-19-related health beliefs were associated with higher intent. This work can inform interventions to increase vaccine uptake, ultimately reducing COVID-19-related morbidity and mortality.

The COVID-19 (coronavirus disease 2019) pandemic, caused by the SARS-CoV-2 (severe acute respiratory syndrome coronavirus 2) virus, emerged in late 2019 with U.S. cases presently at 5.9 million, and >180,000 attributable deaths (Centers for Disease Control and Prevention [CDC], 2020b). With no available vaccine, public health agencies like the Centers for Disease Control have advised the public on specific behaviors to limit transmission (e.g., “social distancing,” wearing a face mask, etc.; CDC, 2020a). Beyond individual behaviors, local and state governments across the country enacted various “stay-at-home” orders and closed nonessential businesses during parts of March, April, and May (Lee et al., 2020). Despite these measures, COVID-19 has caused a serious disease burden to the U.S. health care system. Consensus among medical experts is that until a vaccine is available *and* we reach high-vaccine coverage, nonpharmaceutical interventions will only be able to curb the spread of the virus (Corey et al., 2020).

Several SARS-CoV-2 vaccines are in development and might be available by early 2021, though availability will depend on successful clinical trials demonstrating efficacy and safety (Lurie et al., 2020). Public health and medical practitioners must prepare to promote acceptance of these vaccines. Vaccine hesitancy, which describes a range of stances toward vaccination, from deep skepticism about vaccine efficacy and safety to more mild concerns, has been identified by the World Health Organization as a major global health threat and is particularly prevalent in the United States (MacDonald, 2015; Quinn et al., 2019; World Health Organization, 2019). Because scholars have argued that vaccine hesitancy is driven by context-specific factors including time and place as well as individual factors such as beliefs about threat of disease (Brewer et al., 2007; Dubé et al., 2015; Larson et al., 2014), it is important to understand perceptions related to SARS-CoV-2 vaccination and to assess what factors may contribute to higher or lower intentions to vaccinate.

Previous research with other vaccine-preventable diseases show that there are identifiable factors that may influence vaccination intentions and acceptance. For example, certain sociodemographic factors have played a role in adult vaccination acceptance, such as socioeconomic status, age, race and ethnicity, and geographic location (Abbas et al., 2018; Almario et al., 2016; Galarce et al., 2011). Since vaccination relies on the principle of “herd immunity,” prosocial motives for behaviors that benefit others, such as general altruism, prosociality, and sympathy, can play a role in some vaccination decision making (Li et al., 2016; Vietri et al., 2012). Additionally, theoretical models like the health belief model have long recognized that variables like perceived severity and susceptibility to a disease may predict behavioral intentions, which in turn, predict behavior (Brewer et al., 2007; Bish et al., 2011; Champion & Skinner, 2008; Gerend & Shepherd, 2012; Yang, 2015). The extended parallel process model further posits that health promotion and message design must consider the balance between addressing issues of severity and susceptibility in a way that promotes message acceptance, rather than provoking too much or not enough fear and thus causing people to reject the message (Prati et al., 2012; Quick et al., 2018; Vorpahl & Yang, 2018; Witte, 1992). Vaccine communication and promotion work has long relied on theoretical models like these not only for guiding formative work with target populations (Cameron et al., 2009; Chen et al., 2011) but also to develop and test behavioral interventions (Gerend & Shepherd, 2012; Gore & Bracken, 2005). Finally, research demonstrates that a provider recommendation remains an important predictor of vaccination behavior in the United States (Moss et al., 2016; Reiter et al., 2013). More important, strong provider recommendations are needed to maximize the effect on patient vaccination decisions (Gilkey et al., 2016; Lu et al., 2018).

Given the novel nature of COVID-19, research is needed to assess the public’s intentions to get the SARS-CoV-2 vaccine, when it becomes available, as well as what factors may be associated with higher or lower intent. To ensure high vaccination coverage, public health campaigns must be carefully designed based on evidence about target populations and may even need to employ targeted communication strategies based on sociodemographic and psychosocial variables (Brewer et al., 2017; Dubé et al., 2015, Kriss et al., 2017; Minor et al., 2010; Stockwell et al., 2012). Otherwise, we risk disseminating counterproductive messaging that may reinforce hesitancy in those already hesitant (Bloom et al., 2014). Therefore, a national survey of adults in the United States was used to address the following research questions:

**Research Question 1:** What are the SARS-CoV-2 vaccine behavioral intentions of adults in the U.S.?
**Research Question 2:** What are the SARS-CoV-2 vaccine behavioral intentions of adults in the U.S. when a health care provider recommends the vaccine?
**Research Question 3:** What factors are associated with SARS-CoV-2 vaccine behavioral intentions of adults in the United States?

## Method

### Participants and Recruitment

The data for this study come from a survey assessing knowledge, beliefs, and behaviors related to the COVID-19 pandemic. Data were collected between May 4 and May 11, 2020 through an online survey. Participant recruitment was facilitated by Dynata, a market research firm that maintains panels of 62 million volunteer survey respondents throughout 100 countries. Panelists receive monetary incentives tailored to both the time and effort required for participation and regional preferences. Email invitations were sent to members of Dynata’s U.S. panel who met eligibility criteria of being 18 years or older and able to read English. The study was approved by the university’s institutional review board as exempt and not requiring written informed consent.

A total of 4,042 participants opened the survey and 351 (8.6%) chose not to continue after reading the informed consent welcome page. We excluded anyone who did not answer the intention outcome measures for the current study. Importantly, because vaccine intent and/or need may be different for people who were previously infected with SARS-CoV-2 and perceived threat variables (discussed below) are usually only measured for future threats, only participants who answered “no” to the question “do you believe that you’ve had COVID-19” are included in the current study (*n* = 3,159).

### Measures

In addition to demographic information, the study team collected data on participants’ vaccine behavioral intentions, sociocultural beliefs, experiences with COVID-19, and health beliefs regarding personal risk and threat of COVID-19. Detailed information on variables measured at the categorical level as well as their response options can be found in Table 1; variables measured at the continuous level are described below.

**Table 1.** Sample Description and Bivariate Associations With Overall Vaccine Intent (*n* = 3,159).

Variable	*n* (%) or*M* (*SD*)	^a^Intention to get COVID-19 Vaccine: Means for categorical variables and correlations for continuous variables	Bivariate associations	
			^b^B [95% CI]	Partial η^2^
Demographic characteristics				
Age	46.9 (16.8)	.28	**0.03 [0.03, 0.04]**	0.076
Region				0.002
Northeast	644 (20.6)	5.44	0.09 [−0.12, 0.29]	0.000
Southeast	797 (25.5)	5.38	0.03 [−0.16, 0.22]	0.000
Midwest	686 (21.9)	5.41	0.06 [−0.14, 0.26]	0.000
Southwest	346 (11.1)	5.15	−0.20 [−0.44, 0.05]	0.001
West	657 (21.0)	5.35	Ref.	Ref.
Sex				0.002
Male	1497 (47.2)	5.45	**0.17 [0.04, 0.30]**	0.02
Female	1,657 (52.8)	5.28	Ref.	Ref.
Race/Ethnicity				0.034
Non-Hispanic White	2,039 (65.1)	5.59	**0.57 [0.33, 0.81]**	0.007
Non-Hispanic Black/African American	457 (14.6)	4.66	**−0.36 [−0.64, −0.08]**	0.002
Hispanic	382 (12.2)	5.16	0.13 [−0.16, 0.43]	0.000
Non-Hispanic Other	254 (8.1)	5.03	Ref.	Ref.
Relationship status				0.011
Partnered	1,792 (57.2)	5.54	**0.40 [0.27, 0.54]**	0.011
Not partnered	1,341 (42.8)	5.13	Ref.	Ref.
Children living in home				0.008
No	2,292 (74.5)	5.48	**0.39 [0.24, 0.54]**	0.008
Yes	785 (25.5)	5.09	Ref.	Ref.
Education				0.038
Less than high school graduate, HS graduate, GED	725 (23.2)	4.77	**−0.98 [−1.19, −0.78]**	0.028
Some college/Associate’s degree	899 (28.8)	5.31	**−0.44 [−0.64, −0.25]**	0.006
Bachelor’s degree	923 (29.6)	5.65	−0.11 [−0.31, 0.08]	0.000
Graduate school	572 (18.3)	5.76	Ref.	Ref.
Currently employed				0.020
Yes, full-time (35+ hours per week)	983 (31.4)	5.31	−0.43 [−1.03, 0.17]	0.001
Yes, part-time	439 (14.0)	5.19	−0.55 [−1.16, 0.58]	0.001
Yes, furloughed with pay	75 (2.4)	4.52	**−1.22 [−1.94, −0.50]**	0.004
Yes, furloughed without pay	176 (5.6)	5.58	−0.17 (−0.81, 0.48]	0.000
No, looking for work	348 (11.1)	4.95	**−0.79 [−1.41, −0.18]**	0.002
No, not looking for work	1074 (34.3)	5.63	−0.11 [−0.71, 0.49]	0.000
Other	39 (1.2)	5.74	Ref.	Ref.
Work in health care				0.017
Currently employed in health care	376 (12.2)	4.87	**−0.65 [−0.86, −0.45]**	0.013
Not currently but in the past	453 (14.7)	5.06	**−0.46 [−0.65, −0.27]**	0.007
Never	2260 (73.2)	5.52	Ref.	Ref.
Household income (2019)				0.026
Less than $25,000	985 (32.0)	5.00	**−0.86 [−1.10, −0.63]**	0.016
$25,000-$74,999	959 (31.2)	5.35	**−0.52 [−0.75, −0.28]**	0.006
$75,000-$149,999	821 (26.7)	5.67	−0.20 [−0.44, 0.05]	0.001
$150,000 or more	310 (10.1)	5.86	Ref.	Ref.
Political views				0.008
Liberal	911 (30.7)	5.65	**0.41 [0.24, 0.58]**	0.007
Moderate	11727 (39.5)	5.36	0.12 [−0.04, 0.28]	0.001
Conservative	882 (29.7)	5.24	Ref.	Ref.
Health care characteristics				
Received a flu vaccine, 12 months				0.121
Yes	1,625 (51.7)	5.99	**1.31 [1.18, 1.43]**	0.121
No	1,520 (48.3)	4.69		
Ever received a COVID test				0.008
Yes	229 (7.3)	4.77	**−0.64 [−0.89, −0.39]**	0.08
Result of test				
Positive: 21 (0.7)				
Negative: 175 (5.6)				
Still waiting on results: 29 (0.9)				
No	2,890 (92.7)	5.41	Ref.	Ref.
Preexisting condition that makes COVID-19 more severe				0.016
Yes	1,012 (32.3)	5.71	**0.51 [0.37, 0.65]**	0.016
No	2,121 (67.7)	5.20	Ref.	Ref.
Knows someone who had COVID-19				0.002
Yes, I know someone who had a positive test	706 (22.5)	5.50	0.15 [−0.001, 0.31]	0.001
I believe so; they were sick but unable to get tested/awaiting results	273 (8.7)	5.16	−0.19 [−0.43, 0.05]	0.001
No, I do not know anyone who has been sick with COVID-19	2,152 (68.7)	5.35	Ref.	Ref.
Health belief variables				
High commitment altruism (5 items; range: 1-5)	2.46 (0.91)	.07	**0.15 [0.07, 0.22]**	0.005
Low commitment altruism (4 items; range: 1-5)	3.37 (0.92)	.28	**0.57 [0.50, 0.63])**	0.076
Mean perceived severity of COVID-19 (4 items, range: 1-5)	3.02 (0.88)	.23	**0.49 [0.42, 0.56]**	0.052
Mean COVID-19-related worry (3 items; range: 1 5)	3.47 (1.08)	.40	**0.70 [0.64, 0.75]**	0.162
Likelihood of infection (1 = not at all; 5 = extremely)	2.33 (1.03)	.23	**0.42 [0.36, 0.49]**	0.053
Threat to physical health (1 = not at all; 5 = extremely)	3.06 (1.23)	.29	**0.45 [0.39, 0.50]**	0.085
Believe COVID-19 is major problem in community				0.042
Yes	1,757 (56.3)	5.71	**0.78 [0.65, 0.91]**	0.042
No	1,364 (43.7)	4.93	Ref.	Ref.
Mean likelihood of getting SARS-CoV-2 vaccine without provider recommendation	5.24 (2.0)			
Mean likelihood of getting SARS-CoV-2 vaccine with provider recommendation	5.48 (1.93)			
Overall mean likelihood of getting SARS-CoV-2 vaccine (combined score)	5.36 (1.88)			

*Note. N* = 3,159. Ref. = reference group.

a Overall vaccine intention measure; mean scores for each categorical variable or correlations presented for continuous variables. ^b^Coefficients that are significant at*p* < .05 are in boldface.

#### Vaccine Behavioral Intentions

Two items, adapted from previous vaccine work, assessed participants’ likelihood to receive a SARS-CoV-2 vaccine (Gerend & Shepherd, 2012). Based on pretesting of our survey instruments, it was determined that using the term “COVID-19 vaccine” in the survey was more appropriate for lay audiences, since SARS-CoV-2 is less frequently used in lay communication. These two vaccine intent items included “How likely is it that you’ll get a COVID-19 vaccine, if it becomes available?” (*individual intent*) and “If your healthcare provider strongly recommended a COVID-19 vaccine in the next year, how likely is it that you’d get vaccinated?” (*provider rec intent*). Both items were assessed using a 7-point Likert-type scale (1 = *very unlikely* to 7 = *very likely*). Because these two items were highly correlated with high reliability (Cronbach’s α = .91), the two behavioral intention items were averaged into a single overall intent measure (*overall vaccine intent*).

#### Altruism

We assessed participants’ altruism using an 18-item scale adapted from Rushton et al. (1981). Participants responded to each item on a 5-point Likert-type scale where 1 = *never* to 5 = *very often*. We conducted a principal components exploratory factor analysis, which extracted two factors. We labeled the first factor, which consisted of five items (Cronbach’s α = .83), *high commitment altruism* (i.e., behaviors that require a relatively high level of personal involvement; e.g., “I have helped push a stranger’s car out of the snow or mud.”). We labeled the second factor, which consisted of four items (Cronbach’s α = .81), *low commitment altruism* (i.e., behaviors that require a relatively low level of personal involvement; e.g., ”I have given money to charity.”).

#### Personal Risk and Threat Variables

##### COVID-related worry

A three item scale adapted from Liau et al. (1998) and Fan et al. (2018) was used to measure participants’ personal worry about COVID-19 (“I am scared about getting infected with COVID-19,” “The possibility of getting infected in the future with COVID-19 concerns me,” and “I don’t really worry about getting infected with COVID-19”). Participants responded to each item on a 5-point Likert-type scale where 1 = *strongly disagree* to 5 = *strongly agree*. The last item was reverse coded, and then the three items were summed and averaged to derive a single *COVID-related worry* score (Cronbach’s α = .82).

##### Perceived severity of COVID

A four-item scale adapted from Cahyanto et al.’s (2016) work on Ebola was used to measure participants’ perceptions of the severity of COVID-19 (e.g., “I am afraid that I may die if I contract COVID-19.”). Participants responded to each item on a 5-point Likert-type scale where 1 = *strongly disagree* to 5 = *strongly agree*. The items were summed and averaged to derive a single *perceived severity of COVID* score (Cronbach’s α = .706).

##### Likelihood of infection

Personal susceptibility was measured with a single item: “how likely do you believe it is that you will get infected with COVID-19?” Participants responded on a 5-point Likert-type scale where 1 = *not at all* to 5 = *extremely*.

##### Threat to physical health

Perceived threat to physical health was measured with a single item: “If you got infected with COVID-19, how threatening would it be to your physical health?” Participants responded on a 5-point Likert-type scale where 1 = *not at all* to 5 = *extremely*.

### Analysis

First, the sample was described using frequency distributions or means and standard deviations, as appropriate. We then examined our two vaccination intent variables (*individual intent* and *provider rec intent*) and examined if the participant changed their likelihood of receiving a SARS-CoV-2 vaccine when they were told a provider recommended it.

We then examined bivariate associations between the *overall vaccine intent* score and each of the potential predictor variables using linear regression. Any variable that was significant at *p* < .01 in bivariate linear regression was included in subsequent analyses. We used .01, rather than .05 as the cutoff because, with our large sample size, a cutoff level of .05 might identify trivial relationships. We then conducted a three-step hierarchical multiple linear regression analysis. In the first step, we included demographic characteristics, in the second step we added in health care characteristics, and in the third step we included health belief characteristics. This approach was used to determine if health beliefs influenced likelihood of receiving a SARS-CoV-2 vaccine, above and beyond demographic and health care characteristics.

## Results

### Sample Description

The final analytic sample included 3,159 participants who reported no previous COVID-19 diagnosis. Mean age was 46.9 years (*SD* = 16.8) and the majority of participants were female (*n* = 1,657; 52.8%) and non-Hispanic White (*n* = 2,039; 65.1%). For a complete inventory of sample descriptive statistics, see Table 1.

### SARS-CoV-2 Vaccine Intent (Research Questions 1 and 2)

**Figure 1.**
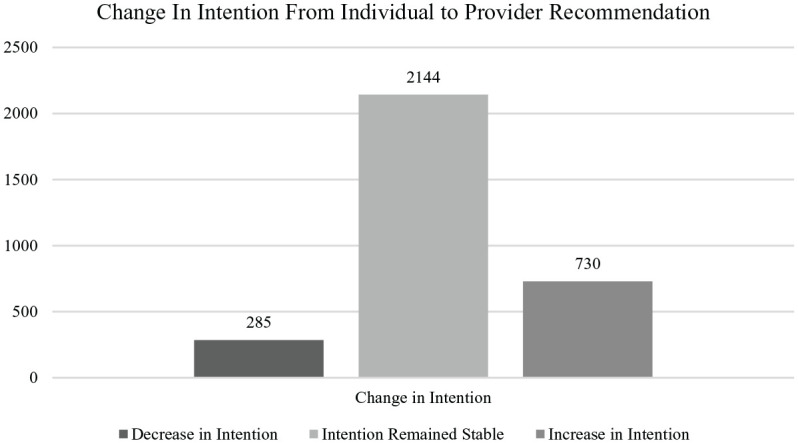
Change in direction of vaccine intent from individual intent to intent with a provider recommendation. *Note. N* = 3,159.

When asked how likely they were to get the SARS-CoV-2 vaccine, the mean score was 5.24 (*SD* = 2.0). This average intention increased to a mean score of 5.48 (*SD* = 1.93) when they were asked the likelihood of receiving the vaccine if their health care provider strongly recommended it. For a categorical breakdown of responses to each of the intent variables, see Table 2. The mean increase from individual intent to provider recommendation intent was significant, *t* = −12.343 (*p* < .0001). When examining change in intent from individual intent to intent due to provider recommendation, the majority of the sample (*n* = 2,144; 67.9%) did not change their response to the likelihood of receiving the vaccine. However, almost one quarter of the sample (*n* = 730; 23.1%) became more likely to receive the vaccine if a provider recommended it and a smaller percentage (*n* = 285; 9.0%) became less likely to receive the vaccine if a provider recommended it; see Figure 1.

**Table 2.** Distribution of Vaccine Intent Measures by Answer Choice.

Intent variable	Likelihood of getting SARS-CoV-2 vaccine without provider recommendation (%)	Likelihood of getting SARS-CoV-2 vaccine with provider recommendation (%)
Very unlikely	8.8	7.5
Somewhat unlikely	5.4	4.0
A little unlikely	4.3	4.0
Neither likely nor unlikely	15.2	13.9
A little likely	9.6	8.8
Somewhat likely	14.7	12.6
Very likely	41.9	49.2

*Note. N* = 3,159.

### Factors Associated With COVID Vaccine Intent (Research Question 3)

In bivariate analyses with overall intent score (individual intent and provider recommendation intent combined; *M* = 5.36, *SD* = 1.88), variables that had associations at *p* > .01 included region (*p* = .207), knowing someone who has had COVID-19 (*p* = .028), and sex (*p* = .013). These variables were not included in subsequent analyses. See Table 1 for all bivariate analyses.

Multivariable regression analyses can be found in Table 3. The first step of the hierarchical multiple regression including only demographic variables that had an adjusted *R*^2^ value of .136. When personal health care variables were added in Step 2, the adjusted *R*^2^ value increased to .220. Finally, in the third step of the hierarchical multiple regression, the adjusted *R*^2^ increased to .318 when the health belief variables were included.

**Table 3.** Multivariable Stepwise Linear Regression.

Variable	Step 1: Demographicvariables		Step 2: Including health care variables		Step 3: Including health belief variables	
	B [95% CI]	Partial η2	B [95% CI]	Partial η2	B [95% CI]	Partial η^2^
Demographic characteristics						
Age	**0.02 [0.02, 0.03]**	0.024	**0.02 [0.01, 0.02]**	0.012	**0.01 [0.01, 0.02]**	0.008
Race/Ethnicity						
Non-Hispanic White	**0.36 [0.11, 0.62]**	0.003	**0.43 [0.19, 0.67]**	0.005	**0.34 [0.11, 0.57]**	0.003
Non-Hispanic Black/African American	−0.15 [−0.44, 0.15]	0.000	−0.03 [−0.32, 0.25]	0.000	−0.09 [−0.36, 0.18]	0.000
Hispanic	0.29 [−0.02, 0.59]	0.001	**0.35 [0.05, 0.64]**	0.002	0.20 [−0.08, 0.48]	0.001
Non-Hispanic Other	Ref.	Ref.	Ref.	Ref.	Ref.	Ref.
Relationship status						
Partnered	0.06 [−0.09, 0.21]	0.000	0.04 [−0.11, 0.18]	0.000	−0.03 [−0.16, 0.11]	0.000
Not partnered	Ref.	Ref.	Ref.	Ref.	Ref.	Ref.
Children living in home						
No	0.06 [−0.10, 0.23]	0.00	0.04 [−0.12, 0.20]	0.000	0.09 [−0.07, 0.24]	0.000
Yes	Ref.	Ref.	Ref.	Ref.	Ref.	Ref.
Education						
Less than high school graduate, high school graduate, GED	**−0.76 [−0.99, −0.53]**	0.015	**−0.62 [−0.84, −0.40]**	0.011	**−0.52 [−0.73, −0.31]**	0.009
Some college/Associate’s degree	**−0.40 [−0.61, −0.19]**	0.005	**−0.31 [−0.51, −0.11]**	0.004	**−0.23 [−0.42, −0.04]**	0.002
Bachelor’s degree	−0.14 [−0.34, 0.05]	0.001	−0.07 [−0.26, 0.11]	0.000	−0.09 [−0.26, 0.09]	0.000
Graduate school	Ref.	Ref.	Ref.	Ref.	Ref.	Ref.
Currently employed						
Yes, full-time (35+ hours per week)	**−0.78 [−1.42, −0.14]**	0.015	−0.47 [−1.09, 0.15]	0.001	−0.23 [−0.82, 0.36]	0.000
Yes, part-time	−0.49 [−1.14, 0.16]	0.001	−0.13 [−0.76, 0.50]	0.000	0.08 [−0.52, 0.68]	0.000
Yes, furloughed with pay	**−1.18 [−1.94, −0.42]**	0.003	−0.68 [−1.43, 0.06]	0.001	−0.44 [−1.15, 0.27]	0.001
Yes, furloughed without pay	−0.41 [−1.09, 0.28]	0.001	−0.09 [−0.75, 0.57]	0.000	−0.05 [−0.68, 0.57]	0.000
No, looking for work	−0.58 [−1.24, 0.08]	0.001	−0.28 [−0.92, 0.36]	0.000	−0.05 [−0.66, 0.56]	0.000
No, not looking for work	−0.55 [−1.18, 0.09]	0.001	**−0.39 [−1.01, −0.22]**	0.001	−0.15 [−0.83, 0.44]	0.000
Other	Ref.	Ref.	Ref.	Ref.	Ref.	Ref.
Work in health care						
Currently employed in health care	**−0.36 [−0.58, −0.14]**	0.004	**−0.45 [−0.67, −0.24]**	0.006	**−0.36 [−0.56, −0.15]**	0.004
Not currently but in the past	**−0.30 [−0.49, −0.11]**	0.003	**−0.33 [−0.51, −0.14]**	0.005	**−0.27 [−0.44, −0.09]**	0.003
Never	Ref.	Ref.	Ref.	Ref.	Ref.	Ref.
Household income (2019)						
Less than $25,000	**−0.51 [−0.78, −0.24**	0.005	**−0.40 [−0.66, −0.14]**	0.003	**−0.31 [−0.56, −0.06]**	0.002
$25,000-$74,999	**−0.25 [−0.50, −0.01]**	0.002	−0.20 [−0.43, 0.04]	0.001	−0.20 [−0.42, 0.03]	0.001
$75,000-$149,999	−0.13 [−0.37, 0.10]	0.000	−0.10 [−0.33, 0.13]	0.000	−0.07 [−0.28, 0.15]	0.000
$150,000 or more	Ref.	Ref.	Ref.	Ref.	Ref.	Ref.
Political views						
Liberal	**0.67 [0.50, 0.84]**	0.021	**0.61 [0.45, 0.78]**	0.020	**0.27 [0.11, 0.43]**	0.004
Moderate	**0.25 [0.09, 0.41]**	0.004	**0.24 [0.09, 0.39]**	0.004	0.10 [−0.04, 0.25]	0.001
Conservative	Ref.	Ref.	Ref.	Ref.	Ref.	Ref.
Health care characteristics						
Received a flu vaccine, 12 months						
Yes			**1.09 [0.96, 1.22]**	0.091	**0.90 [0.77, 1.02]**	0.071
No			Ref.	Ref.	Ref.	Ref.
Ever received a COVID-19 test						
Yes			−0.41 [−0.67, −0.15]	0.004	−0.24 [−0.49, 0.01]	0.001
No			Ref.	Ref.	Ref.	Ref.
Preexisting condition that makes COVID-19 more severe						
Yes			**0.25 [0.11, 0.38]**	0.005	−0.10 [−0.25, 0.05]	0.001
No			Ref.	Ref.	Ref.	Ref.
Health belief variables						
High commitment altruism (5 items; range: 1-5)					−0.04 [−0.12, 0.05]	0.000
Low commitment altruism (4 items; range: 1-5)					**0.19 [0.11, 0.28]**	0.007
Mean perceived severity of COVID (4 items, range:1 -5)					−0.07 [−0.17, 0.03]	0.001
Mean COVID-19-related worry (3 items; range: 1-5)					**0.43 [0.36, 0.51]**	0.047
Likelihood of infection (1 = *not at all*; 5 = *extremely*)					**0.07 [0.00, 0.14]**	0.002
Threat to physical health (1 = *not at all*; 5 = *extremely*)					**0.11 [0.04, 0.18]**	0.004
Believe COVID-19 is major problem in community						
Yes					**0.21 [0.08, 0.35]**	0.004
No					Ref.	Ref.

*Note*. Step 1 *R*^2^ = .143 (adjusted = .136); Step 2 *R*^2^= .227 (adjusted = .220); Step 3 *R*^2^= 0.327 (adjusted = .318). Backward selection with *p* < .01 to stay. Removed partnership status (*p* = .702), high-commitment altruism (*p* = .392) children living in home (*p* = .205), preexisting condition (A1.04, *p* = .219), likelihood of infection (A1.02, *p* = .055), severity (*p* = .080), employment status (*p* = .033), income (*p* = .076), received a test to check for COVID-19 (A1.05, *p* = .019). The final model has an *R*^2^ = .320 (adjusted = .316). Not significant at .01 in bivariate comparisons: region (*p* = .207), knows someone who’s had COVID-19 (*p* = .028), and sex (*p* = .013). Ref. = reference group. Boldface type indicates statistical significance (*p*<0.05).

In Step 3 of the hierarchical regression model, with all variables included, less education was associated with lower intent to receive a SARS-CoV-2 vaccine. Likewise, being currently employed in health care was also negatively associated with intent to receive a vaccine as compared with those who were never employed in the health care system (Β = −0.36; 95% CI [−0.56, −0.15]). Participants who self-identified as liberal reported the highest intent to receive a SARS-CoV-2 vaccine (Β = 0.27; 95% CI [0.11, 0.43]), followed by moderates, and then conservatives. The health belief variables that were significant in the full regression model were all positively associated with intent to receive a SARS-CoV-2 vaccine. Specifically, as low-commitment altruism increased, likelihood of receiving a SARS-CoV-2 vaccine increased (Β = 0.19; 95% CI [0.11, 0.28]). Furthermore, as perceived threat to physical health increased, likelihood of receiving a SARS-CoV-2 vaccine increased (Β = 0.11; 95% CI [0.04, 0.18]). Those who believed COVID-19 was a major problem in their community had higher likelihood of receiving a SARS-CoV-2 vaccine compared with those who did not (Β = 0.21; 95% CI [0.08, 0.35]). Worry was most strongly associated with SARS-CoV-2 vaccine intent; as worry increased, intent likewise increased (Β = 0.43; 95% CI [0.36, 0.51]).

## Discussion

This article aimed to examine U.S. respondents’ intentions to receive the SARS-CoV-2 vaccine when it becomes available, and investigate factors associated with those intentions. Overall, participants in this study reported high intentions to receive a SARS-CoV-2 vaccine, which were even higher with a strong provider recommendation. Several sociodemographic and health belief variables were also associated with higher and lower SARS-CoV-2 vaccine intentions. Below, we discuss the implications of these findings and suggest areas for future work, including research and practical application.

### High Vaccine Intentions

Importantly, participants reported relatively high individual intent to receive a SARS-CoV-2 vaccine. On a 7-point scale, participants in this study reported an average of 5.23. While not quite a ceiling effect, we believe this suggests strong support for a vaccine, more so because no vaccine has been fully tested and made available to the public. Our findings are consistent with other recent work examining perceptions of the SARS-CoV-2 vaccine, also showing high-vaccine intentions in the United States (Reiter et al., 2020; Thigpen & Funk, 2020). Interestingly, this level of intention to receive the SARS-CoV-2 vaccine is markedly higher than what is seen for actual U.S. adult vaccination behaviors for influenza. The CDC reports that 2018-2019 flu vaccination coverage among adults ≥18 years was only 45.3% (CDC, 2019). Related, research shows that the relationship between intention and actual behavior, while usually significantly positive, is not always a perfect correlation and that different predictors (e.g., perceived susceptibility, doctor recommendation) may differently predict intentions versus actual behavior (Juraskova et al., 2011; Krawczyk et al., 2012; Schwenk & Möser, 2009; Webb & Sheeran, 2006). Therefore, while participants in this study expressed high SARS-CoV-2 vaccine intentions, these findings should be interpreted cautiously. Actual uptake of a future vaccine will likely depend on many factors, including the status of the COVID-19 pandemic at the time of vaccine debut.

Of note for communication scholars, these findings suggest that social normative messaging could capitalize on the high level of vaccine intention. Social norms campaigns use descriptive norms (i.e., descriptive statistics) to correct or reinforce the frequency with which others are performing a behavior, with the assumption that individuals seek to conform to the pressures of societal norms (i.e., subjective norms; Burchell et al., 2013). While most social norms campaigns target audiences who may be overestimating the frequency of an unhealthy behavior (e.g., binge drinking; Campo et al., 2004), the same normative principles have been found to significantly predict HPV vaccination intentions and uptake among young women (de Visser et al., 2011). For example, social norms messages can address SARS-CoV-2 vaccine hesitancy by highlighting the high intentions to vaccinate expressed by the majority of people in one’s social network. This approach will require communication scientists to engage in formative research to develop and test messages with different audiences, especially given the differences in intention across subgroups of population found in this study.

### Provider Recommendation Makes a Difference

Participants in this study also were significantly more likely to receive the vaccine if their health care provider strongly recommended it. This finding is consistent with previous work showing a doctor’s recommendation is a significant predictor of vaccination behavior (Gorman et al., 2012; Rahman et al., 2015; Sturm et al., 2017), including when newer vaccines, such as the 2009 H1N1 influenza vaccine, are being considered (Coe et al., 2012). A key limitation of this study is that the single-item measure only asked participants about intentions if their provider strongly recommended the vaccine; no information was gathered about what information they may want about the SARS-CoV-2 vaccine from their provider.

Providers are the most trusted source of health information for patients (Jackson et al., 2019), including information about vaccines (Eller et al., 2019), which may be important once a SARS-CoV-2 vaccine becomes widely available. Vaccine promotion campaigns may need to emphasize the importance of talking with a health care provider about the vaccine, including asking for information to address any concerns or questions. At the same time, health care providers may need support and training such as that already offered through the CDC (CDC, 2016; CDC, 2018) to be most effective in recommending a SARS-CoV-2 vaccine.

### Factors Associated With Intention

Specific sociodemographic and health belief variables were associated with intentions to vaccinate, and are worthy of consideration for future work, especially for communication interventions seeking to promote a SARS-CoV-2 vaccine.

#### Demographics

Participants with less education expressed a lower intention to receive a SARS-CoV-2 vaccine. Education is often associated with health literacy (Kutner et al., 2006; Paasche-Orlow et al., 2005), suggesting the critical importance of educating the public on the role of vaccines in reducing COVID-19 prevalence through herd immunity. These efforts may need to be done in conjunction with messages about how herd immunity works, as previous work has shown that limited understanding can undermine vaccination intentions and behavior (Sobo, 2016). The effective deployment of “flatten the curve”—a phrase previously not commonly used among lay audiences when discussing a disease outbreak—via social media is an example of effectively educating the public about complex health terms in accessible ways (Boboltz, 2020).

Interestingly, participants who were employed in health care indicated a lower vaccine intention. This was contrary to what was expected. Previous work has shown that some health care providers express vaccine hesitancy and low-vaccine acceptance themselves (Collange et al., 2016; Verger et al., 2015). Additionally, our question only queried whether the individual worked in health care and did not distinguish positions entailing direct patient care or type of training. Given that many health care-related positions are nonclinical (e.g., janitorial, receptionist), some participants who answered this question may have limited understanding about the role of vaccines in preventing infectious diseases. We believe further work is needed to clarify this finding.

Participants’ self-reported political views were associated with vaccine intent, with liberals expressing the strongest SARS-CoV-2 vaccine intentions, followed by moderates, and then conservatives. The United States has a complex and often partisan political environment, which may be compounded by mass media news consumption and “echo chambers” within social media platforms (Bakshy et al., 2015; Iyengar & Hahn, 2009). One group espousing significantly lower intentions than other groups represents a potential challenge for high vaccine community coverage; however, these media trends may also represent an arena for targeted messaging going forward. We make an especially strong call for future work on this issue and implore other health and science communication researchers and practitioners to devote particular attention to targeted work on political ideology as we inch closer to an available SARS-CoV-2 vaccine.

Finally, we found that as individuals’ level of low commitment altruism increased, so too did their likelihood of receiving a SARS-CoV-2 vaccine. Importantly, we all must remember that vaccines provide both a personal benefit and public health benefit. Research on the relationship between concepts like altruism and vaccination is an area that has received increasing, but still inadequate, attention in the vaccine literature (Korn et al., 2020; Li et al., 2016; Quadri-Sheriff et al., 2012; Vietri et al., 2012). Going forward, research examining individual’s concern for the “other” as a potential motivating factor for SARS-CoV-2 vaccination, as well as a potential message design strategy, is an important focus.

#### Perceived Threat and Fear of COVID-19 Associated With Higher Vaccine Intentions

Consistent with frameworks like the health belief model and the extended parallel process model, individuals who expressed fear—measured in this study as higher worry, perceived threat to physical health, and perceived COVID-19 to be a major problem in their community—were more likely to intend to get the SARS-CoV-2 vaccine when it becomes available. The data for this study were collected in early May 2020, when many states in the United States were still in “lock down” mode and COVID-19 rates and hospitalizations were high but steady. If COVID-19 rates and hospitalizations are high when the vaccine debuts, these perceived threat variables may continue to be positively associated with intention. However, if infection rates drop or individuals become numb to the threat posed by the disease, these variables may not be as strongly associated with intentions. It will be important, therefore, to do both longitudinal and cross-sectional surveys over time to monitor changes in public attitudes and perceptions about COVID-19 disease and a SARS-CoV-2 vaccine as well as examine the potential association of other social and behavioral determinants of health such as access and cost issues. In the meantime, communication scientists can capitalize on these findings by exploring messaging strategies that address individuals’ fears about COVID-19.

### Limitations

A limitation of this study is that we used a national but not a population representative sample. Participants were members of an opt-in panel and may not reflect all U.S. adults. Furthermore, the cross-sectional survey design precludes determination of causal direction in the relationships identified and necessarily represents a snapshot in time, rather than the evolving landscape of the public’s knowledge and attitudes about COVID-19. As previously noted, intent can be an imperfect predictor of subsequent behavior. Finally, two measurement limitations worth mentioning include a mismatch in the wording of our intention measures (i.e., the provider intention measure specified a timeline of “in the next year” while the individual intention item did not) and excluding participants who believed they had a previous SARS-CoV-2 infection from the health belief items (e.g., perceived severity, worry, likelihood of infection, threat).

### Conclusions

This study examined SARS-CoV-2 vaccine intentions and factors associated with these intentions. In addition to high intentions to receive the vaccine, provider recommendation increased intentions and will likely be an important factor in achieving the level of vaccination needed for herd immunity. Several sociodemographic and health belief variables were associated with vaccine intentions and suggest important targets for future health and science communication to both educate and promote uptake of a SARS-CoV-2 vaccine. When a vaccine (or vaccines) become available for the public, we must use evidence-based strategies for designing our educational and promotional messaging. The current study provides a starting point for SARS-CoV-2 vaccine communication research in the United States.
